# Health Tract, No. 121

**Published:** 1862-11

**Authors:** 


HEALTH TRACT, No. 121.

### THE DEAF HEAR.

Some become deaf in very early life, in consequence of an unfavorable recovery from scarlet fever, measles, mumps, and other ailments, such as cold in the ears, or by the violent straining of vomiting. Others grow deaf as a consequence of increasing age. In all these the deafness grows with advancing years. A great multitude of remedies have been tried for the removal or mitigation of this calamity; but with the exception of such cases as are the result of "hardened wax," the writer has never known any material benefit to have been derived in a single instance, either by medicines or external appliances. The successful cases were the result of moist bland applications, of which glycerine is the best, from the quality which it possesses of remaining moist longer than any other known substance. Let fall two or three drops in the ailing ear, then introduce a bit of lint or cotton saturated with it. If by repeating this operation night and morning, for some weeks, there is no relief, it may be considered a remediless infirmity. But the increase of the deafness will be considerably retarded, by using all possible means to keep up the general health, by regular bodily habits, by personal cleanliness, by a temperate life, and by arranging to spend several hours of each day in the open air, in some enlivening and agreeable manner.

Artificial aids have sometimes been called into requisition; such as ear-trumpets and auricles, which never fail to deepen the deafness, and that rapidly. It is therefore wisest and best for one who hears with difficulty,

1. To apply glycerine, night and morning, for months.
2. Maintain a high state of general health.
3. Steadily resist all artificial aids for the ordinary occasions of life.
4. Never allow any thing stronger than sweet-oil, tepid water, or glycerine to be applied to the ear.
5. Never permit the introduction of a probe or stick, or any thing else into the ear, for any purpose whatever.

In one case art is admissible—that is, in religious worship; and this being only once or twice a week, the hearing will not be appreciably impaired in the course of several years.

The writer knows a lady who has not heard a sermon for several years, although a regular attendant. She now hears with the utmost ease. This has been accomplished by a peculiar arrangement of that part of the pulpit on which the Bible is laid, and a distribution of pipes under the floor and through the pew-seat. The sound of the speaker's voice can be transmitted with perfect distinctness to various parts of the house, without appreciably affecting the volume of sound—that is, an apparatus arranged for one person, enables him to hear with perfect clearness; if extended to a dozen others, the first one hears as well as if there was but a single attachment. To Christian men and women whose hearing is defective, and who are thereby cut off from one of the greatest privileges of life, this device is of inestimable value; for as we grow old, and the ties which bind us to the world become almost daily fewer and more fragile, we instinctively draw closer to Him who has appointed religious worship as a means of communicating to us his will. Those communications become sweeter, more nourishing, and more necessary every day to the ripe and aged Christian; they are the greatest solace in life. Thus it is he feels with King David, "a day in thy courts is better than a thousand" any where else. Single attachments are made for fifty dollars, and at much less cost when there are two or more in one church, by application to D. D. Stelle & Co., 85 Leonard St., New-York City, who have not failed in a single case, thus far, to give entire satisfaction, by their PHONOPHORUS or *Sound Conductor*.

